# Distinguishing Dependent-Stage Secondary Epileptogenesis in a Complex Case of Giant Hypothalamic Hamartoma With Assistance of a Computational Method

**DOI:** 10.3389/fneur.2020.00478

**Published:** 2020-06-10

**Authors:** Zhao Liu, Guoming Luan, Chuanzuo Yang, Yuguang Guan, Changqing Liu, Jing Wang, Mengyang Wang, Qingyun Wang

**Affiliations:** ^1^Department of Functional Neurosurgery, Sanbo Brain Hospital, Capital Medical University, Beijing, China; ^2^Beijing Key Laboratory of Epilepsy, Epilepsy Center, Sanbo Brain Hospital, Capital Medical University, Beijing, China; ^3^Beijing Institute for Brain Disorders, Beijing, China; ^4^Department of Dynamics and Control, Beihang University, Beijing, China; ^5^Department of Neurology, Sanbo Brain Hospital, Capital Medical University, Beijing, China

**Keywords:** stereo-electroencephalography, refractory focal epilepsy, epileptogenic zone localization, coupled neuronal population model, epileptogenic networks, hypothalamic hamartoma

## Abstract

Besides gelastic seizures, hypothalamic hamartoma (HH) is also noted for its susceptibility to remote secondary epileptogenesis. Although clinical observations have demonstrated its existence, and a three-stage theory has been proposed, how to determine whether a remote symptom is spontaneous or dependent on epileptic activities of HH is difficult in some cases. Herein, we report a case of new non-gelastic seizures in a 9-year-old female associated with a postoperatively remaining HH. Electrophysiological examinations and stereo-electroencephalography (SEEG) demonstrated seizure onsets with slow-wave and fast activities on the outside of the HH. By using computational methodologies to calculate the network dynamic effective connectivities, the importance of HH in the epileptic network was revealed. After SEEG-guided thermal coagulation of the remaining HH, the patient finally was seizure-free at the 2-year follow-up. This case showed the ability of computational methods to reveal information underlying complex SEEG signals, and further demonstrated the dependent-stage secondary epileptogenesis, which has been rarely reported.

## Introduction

As a rare congenital malformation disease, hypothalamic hamartoma (HH) has four major impacts on patients, especially in the pediatric populations: precocious puberty (PP) (1), seizures that are mainly gelastic seizures (GS) (2), cognitive and behavioral impairments (3), and developmental delays (4). Among the symptoms, GS is a hallmark, mostly drug resistant and is verified by stereo-electroencephalography (SEEG) as originating from HH (5, 6). However, due to the observation of multiple other seizure types associated with extra-lesion areas, a hypothesis of secondary epileptogenesis, that persistent seizure activities from HH could induce seizure activities in various neocortical areas, has been suggested (5, 7). According to Morell's postulation, secondary epileptogenesis develops in three stages (i.e., dependent, intermediate, and independent) (8, 9). Previous clinical observations have demonstrated the existence of the independent stage, but methods to distinguish this stage once the secondary epileptogenesis emerges have rarely been described (7). Since the 1990s, computational methods based on various theoretical models have been developed and used to solve problems that were too complex for manual interpretations (10–14).

In this paper, we report a giant (diameter > 5 cm) HH case, whose epilepsy control did not merely fail after a secondary-stage surgery approach, but secondary epileptogenesis also developed. Despite the difficulty of diagnosing the stage of secondary epileptogenesis with information from magnetic resonance imaging (MRI) scans, scalp video electroencephalograms EEGs, and SEEG, use of a novel computational method based on SEEG data suggested the possibility of a dependent stage, and the patient's seizure control finally succeeded with SEEG-guided thermal coagulation.

## Case Presentation

**Figure 1:**
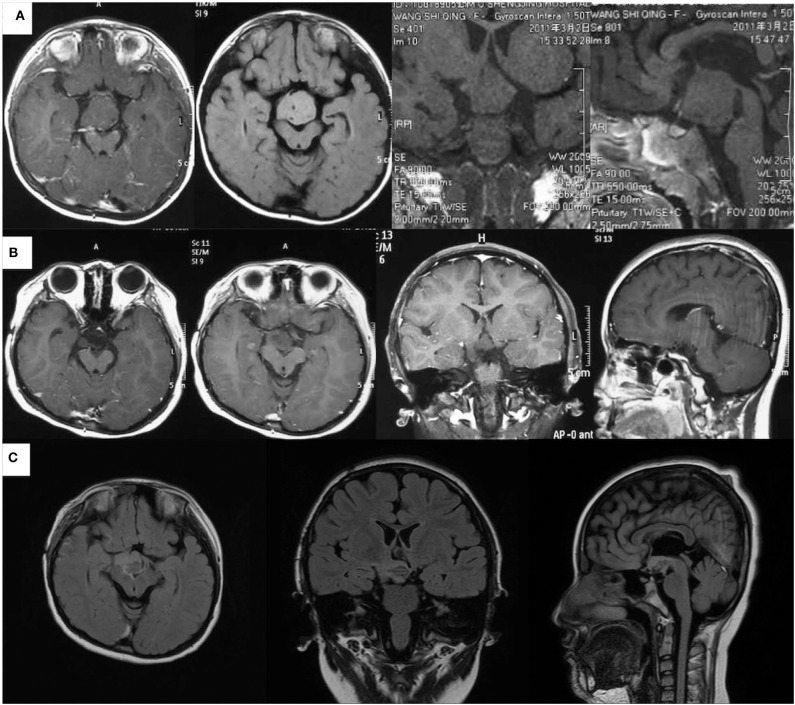
Magnetic resonance imaging scans of the patient at different ages. **(A)** Images at 2.5 years of age before the first resective operation the giant HH (about 35 × 32 × 25 mm, images from left to right are the axial contrasted T1 image, axial T2 FLAIRE image, coronal T1 image, and sagittal T1 image). **(B)** Images at 3.5 years of age after the second resective operation demonstrated about 25% of the lesion had remained (images from left to right are two axial contrasted T1 images in different slices, contrasted coronal T1 image, and contrasted sagittal T1 image). **(C)** Images 7 days after the ablation showed that almost all the par connection between the HH and the hypothalamus has been coagulated. (Images from left to right are axial, sagittal, and coronal T2 FLAIRE images).

A 9-year-old female was admitted to our epilepsy center because of an almost 8.5-year history of compulsive bursts of giggles, which probably started since 8 months of age. At that time, giggles were not confirmed because the sounds merely sounded like a peculiar noise. A giant HH, which was approximately 35 × 32 × 25 mm had grown to the interpeduncular cistern as revealed by an MRI scan (Figure 1A). GS were diagnosed at 2 years of age, and two resective operations had been conducted separately at 6 months and 1.5 years later. Both operations were via the same trans-right-frontal-basal approach and about 50% of the lesion had been removed (Figure 1B), and pathological examinations verified the diagnosis of HH. After the first operation, carbamazepine therapy of 100 mg per day was given. Unfortunately, the two operations and medical therapy did not improve the compulsive giggles. Later, since 7 years of age, carbamazepine had been ceased by her mother without medical consultation. The patient had no antiepileptic drugs and her GS had not changed until her admission to our center due to a recent aggravation with two times of a new seizure type. These two seizures started with a loss of consciousness and then developed to her eyes turning to the left and then tonic–clonic seizure of the four limbs. This lasted for about 2–3 min. The patient had medium-level academic achievement, and her mother reported no obvious behavioral deterioration. Nevertheless, the patient was described as short-tempered and hard to communicate with.

Her physical and neurological examinations were normal. The Wechsler Intelligence Scale for Children—Chinese Revised showed that her full intelligence quotient was at an average level with a score of 97. All routine blood, blood coagulation, and biochemical tests, as well as infection immunoassay results, urinalysis, electrocardiogram, and chest radiography showed normal results.

For epilepsy evaluation, both scalp video-EEG (VEEG) and MRI scans were conducted. Structural imaging showed that the remaining HH was connected to the hypothalamus (Figure 1B).

VEEG were recorded with a Nicolet video-EEG monitoring system (Thermo Fisher Scientific, Waltham, MA, USA) and digitized at the rate of 1,024 Hz with the international standard 10–10 electrode montage. The online band-pass filter was set to 1.6–150 Hz. The monitor recorded for 7 days and video observations demonstrated two types of clinical seizures. The first was the bursts of giggles, which persisted for about 5 s, and the second one was the loss of consciousness followed by head-turning to the left, which lasted about 20 s. No obvious giggles were observed during the second procedure, and associated auras were denied.

**Figure 2:**
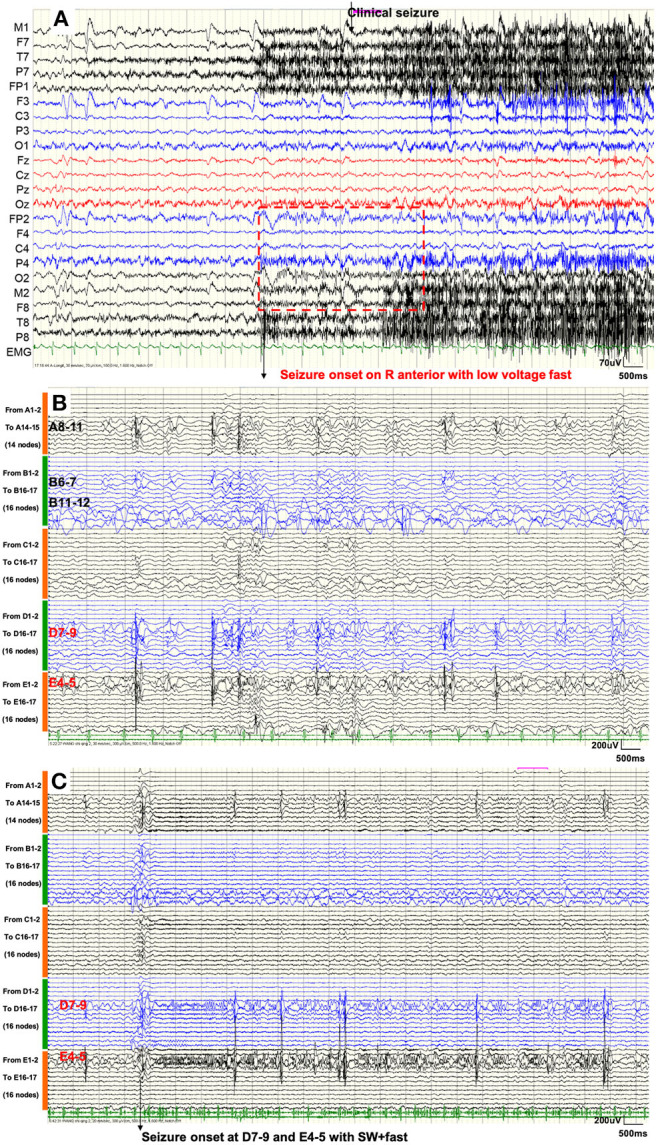
**(A)** Scalp VEEG showed seizure onset with the consciousness loss with head-turning to the left was located on the right anterior area with low-voltage fast activities. **(B)** SEEG demonstrated that inter-ictal discharges emerged only within the temporal lobe (nodes A8–11, B6–7, B11–12, D7–9, and E4-5). **(C)** SEEG showed that GS started within the right hippocampus (nodes D7–9 and E4–5) with spike–waves in fast activities. Seizure activities were not recorded, either during the inter-ictal period or during the seizure procedure, in nodes within the remaining HH.

In the inter-ictal period, intermittent poly-spikes and slow-wave activities were recorded in the right frontal area (F8, Fp2, F4) and the right temporal area (M2, T4). In the peri-ictal period, the EEG onset zone was located in the right hemisphere and was obvious in the anterior area (Fp2, F4, C4, M2, F8, T8) with low-voltage fast activities. Two seconds later, the clinical symptoms started. Figure 2A shows the VEEG waveforms of 6 s pre-ictal and 11 s early-ictal period of the second seizure type. The GS showed similar VEEG performances.

From these data, three hypotheses for the secondary partial seizures emerged; these shared the same mechanisms with GS induced either by HH or some region in the neocortex. Likewise, it might independently oscillate similar to VEEG performances with GS induced by HH.

To accurately explore the seizure onset zone, five intracranial electrodes were stereotaxically implanted with a robot-assisted stereotaxic operation system (ROSA). The SEEG depth electrodes (16 contacts, length: 2 mm, diameter: 0.8 mm; 1.5 mm apart) were manufactured by ALICS Co Ltd., Besancon, France. The diameter of the depth electrode was 0.8 mm. The electrodes were placed into the remaining HH via the right anterior temporal lobe (electrodes A–D) or right anterior frontal lobe (electrode E). The SEEGs were recorded using a common reference electrode (Nicolet™ system; 128 channels; sampling rate, 1024 Hz). The impedance of all the recording electrode nodes was kept below 50 kΩ; otherwise, the nodes would be excluded from analyses. Bipolar derivation was chosen to avoid possible bias deriving either from a not completely inactive common reference or from interference due to a volume conduction effect. To verify the correct placement of the electrodes, a postimplantation (DynaCT; Siemens, Malvern, PA, USA) scan was performed and reconstructed images were digitally fused with the presurgical MRI dataset using the fusion system within ROSA.

**Figure 3:**
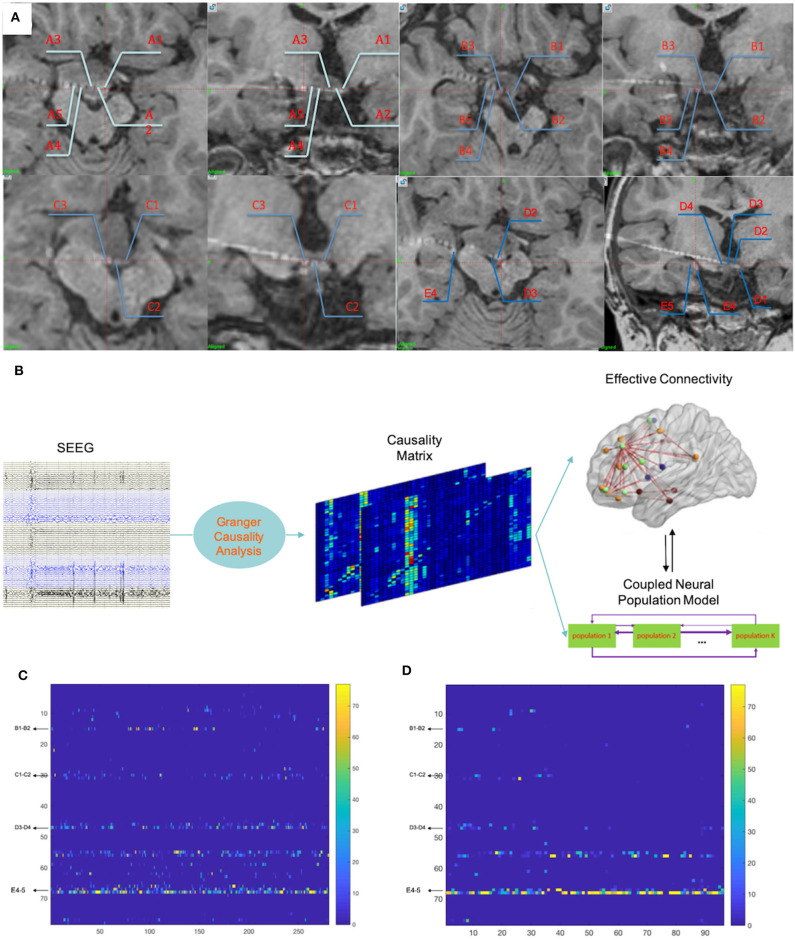
**(A)** Fusion images of CT and T1 images demonstrated locations of the electrode nodes. Nodes A1–5, B1–5, C1–3, and D1–4 were located within the remaining HH. E4–5 were located in the right hippocampus that occurred during implantation. **(B)** The whole procedure of the computational method. More details have been described in a previous study (10). **(C)** The results of calculations of the 5 min SEEG data before seizure onset demonstrated that nodes within the remaining HH (nodes B1–2, C1–2, and D3–4) also contained prominent out-degrees, while nodes within the hippocampus (nodes E4–5) contained dominant out-degrees. **(D)** The results of calculations of the 90 s ictal data showed dominant out-degrees within the hippocampus (nodes E4–5). Numbers in the vertical axis correspond to nodes of the electrodes: 1:14 = A1–2:A14–15; 15:29 = B1–2:B15–16; 30:45 = C1–2:C16–17; 46:61 = D1–2:D16–17; 62:77 = E1–2:E16–17.

Finally, electrodes A–D were implanted as planned while the tip of electrode E was placed into the hippocampus. As a result, electrode nodes A1–5, B1–5, C1–3, and D1–4 were located within the remaining HH; nodes A8–11, B7–9, D7–10, and E4–5 were located within the hippocampus; nodes A14–15, B12–17, C14–17, and D15–17 were located within the right superficial temporal lobe; nodes E16–17 were located within the right superficial frontal lobe; and the other nodes were located within the white matter. Figure 3A shows all nodes within the remaining HH. SEEGs were monitored for 3 days after implantation, and a total of four seizures in two types were captured.

Figure 2B shows that SEEGs for all the inter-ictal discharges were located within the temporal lobe (A8–11, B6–7, B11–12, D7–9, and E4–5). Unexpectedly, seizure onsets of the two types, even the GS, originated from the hippocampus (E4–5 and D7–9) with spike–waves in fast activities as shown in Figure 2C. The time intervals between onset activities and clinical symptoms (giggles or head-turning) were about 14 s.

Because this diverged from clinical experiences, that GS were mostly induced by electrical/physiological activities of HH, the SEEG data were further analyzed by a hemi-manual computational method, which was previously introduced (10; in Matlab 2017). This computational method calculated the SEEG dataset in all bands. Using the adaptive direct transfer function, the SEEG data were integrated into a frequency domain. We then essentially used the Granger causality technique (15) and a time-variant autoregressive model to evaluate the statistical interdependence of multiple simultaneous time series, considering the Kalman filtering algorithm (16). Each node stood for its adjacent neural population. Finally, the instant out-degree of each node in a network was drawn, which stood for the impact of the node on other neural ensembles in the production of synchronous discharges. Figure 3B shows the analyzing process; more details have been described in our previous study (10). For this case, two time periods, which were 5 min before (pre-ictal) and 90 s after (early-ictal) seizure onset, were analyzed, as these datasets could reflect neural network evolution during seizure activities. Thereafter, prominent out-degrees within HH (B1–2, C1–3, and D2–4) were demonstrated in the pre-ictal period (Figure 3C), although the hippocampus (E4–5) persistently showed dominant out-degrees.

From these data, our hypothesis was that the patient showed a dependent-stage elevated secondary epileptogenesis, and therefore, radiofrequency ablation therapy targeting the remaining HH was designed. The parameters are listed in Table 1.

**Table 1:** Parameters of SEEG-guided radiofrequency coagulation.

**Ablation nodes**	**Power rate (W)**	**Impedance pre-abl. (Ω)**	**Impedance post-abl. (Ω)**	**Ablation time(s)**
A (1,2)	3.5	840	750	120
A (2,3)	3.5	800	760	120
A (3,4)	3.5	870	750	120
A (4,5)	3.0	923	800	30
B (1,2)	3.5	615	470	60
B (2,3)	3.5	590	440	60
B (3,4)	3.5	>1000	610	60
B (3,4)	3.0	690	485	30
C (1,2)	3.0	950	900	60
C (2,3)	3.0	960	900	60
A2–B1	3.5	707	670	60
A3–B1	3.5	750	600	60
A4–B2	3.5	890	-	Termination^*^
B1–C2	3.5	850	800	60

*The ablation times and power rate were set according to location of the nodes. When the nodes were close to hypothalamus, a lower rate (3.0 W) with lower time (30 s or 60 s) would be chosen*.

* *The ablation of A4–B2 was terminated because of the intolerance headache*.

Of note, when seizures ceased right after the ablation, post-ablation SEEG monitoring recorded an abrupt decrease of inter-ictal discharges. In addition, the patient had a curative feeling, and the parent described a personality change in the patient as becoming gentler and easier to communicate with. Post-ablation MRI showed a satisfying ablation of the par connection between the HH and the hypothalamus (Figure 1C). After the ablation, an oxcarbazepine therapy of 600 mg per day was given as the postoperative antiepileptic drug. At the 2-year follow-up, there was no sign of relapse and VEEGs (four times for 16 h) showed continued “running down” of inter-ictal discharges. The oxcarbazepine was gradually withdrawn to 300 mg per day. No intelligence decline and behavioral deterioration was observed and the change of personality seemed to be permanent.

## Discussion

In this case, the existence of the dependent stage of secondary epileptogenesis was first suggested. Secondary epileptogenesis was defined by Morell as the involvement of a previously normal neural network by an interconnected actively discharging an epileptogenic area (9), which might be related to the kindling procedure (17). Three stages were postulated: the dependent stage, the intermediate stage, and the independent stage (8). When driven by the primary focus, epileptic activities could be ceased after exclusion of the primary focus in the dependent stage. After temporary persistence, secondary epileptic activities would finally cease after the removal of the primary focus. However, in the dependent stage, in spite of removal of the primary focus, secondary epileptogenesis epileptic activities may persist. A kindling phenomenon, which was described by Goddard (17), was believed to be the cause. Repetition of kindling-like seizure activities may recruit uninvolved neural populations into epileptic networks and the rate of kindling-like activities may decide the secondary-epileptogenesis stage. However, because of the controversy regarding the definition, and the imperfect fit of animal models to the human epileptic syndrome, “secondary epileptogenesis” remained controversial except in the HHs.

Kindling-like activities have been revealed in human HH tissues. In a series of pathological researches, it was recognized that clusters of 80–90% HH neurons, which have an interneuron-like phenotype, work as a pacemaker and the other HH neurons, which are large cells with pleomorphic soma and dendrites, function as a neurotransmitter (18–21). These activities could propagate to the temporal lobe through the left fornix (22) or to the frontal lobe through the mammillary-thalamo-cingulate pathway (23). Scholly et al. have reported HH cases consistent with the independent stage (5, 7). Parvizi et al. suggested that the development of non-GS types in GS with HHs correlates with older age and longer duration of epilepsy (24), while GS related to frontal, parietal lobe epilepsy or hippocampal sclerosis has also been reported (25). Several reports suggested that seizure types besides GS in HHs were related to neocortical seizure activities (5, 26).

In the current case study, the cessation of the seizures, especially non-gelastic seizures after thermal coagulation of par connection and no relapse in the 2-year follow-up, strongly suggested that these seizure activities correlated with the remaining HH. Although electrophysiological examination, especially SEEG, could not directly confirm this correlation, and the SEEG directly indicated seizure onset in the right hippocampus (E4–5, D7–9) (Figure 2C), it might be induced by a lack of HH tissues and the insufficient sensitivity of the SEEG equipment. As previously mentioned, the seizure activities of the HHs were generated by the intrinsic pacemaker neurons (18–21). In this case, the majority of the HH was excluded. The remaining volume of the HH may not generate potentials that could be recorded. The maximum frequency of our SEEG equipment was 1,024 Hz, and it was therefore probable that high-frequency neural oscillations could not be recognized. There was also a window period for anti-epileptic drug treatment, and oxcarbazepine was added after the thermocoagulation. The medication therapy was not believed to play a dominant role, because carbamazepine was not reported to work on seizure control. Considering all these factors, there was a high probability that the patient was in the dependent stage of secondary epileptogenesis, which has seldom been reported.

In similar clinical processes, recognizing the correlation between dependent-stage secondary epileptogenesis and HH preoperatively is crucial to prevent damages from surgical interventions. Computational methods might provide perspectives beyond the manual interpretation of SEEG data.

In recent years, different computational methods have been widely applied to epilepsy clinical studies. Sinha et al. have developed a simulated resection method for neurosurgical outcome prediction based on calculating the escape time, which indicates the possibility of a normal neural population generating abnormal behavior (27). Bartolomei et al. have developed the epileptogenicity index calculation (EI) method, which considers the intensity and frequency of unit neural ensemble activity, to determine the epileptogenic zone (13) Using this method, Scholly et al. analyzed SEEG data of a HH patient (5). The method applied in this study considered the interactive influence of neural populations, which was indicated by the out-degrees of each node, and the dynamical alteration of a seizure network. This was the main strength of this approach. In our previous research (10), the accuracy of our computational method for epileptogenic zone location was 82.86%, and the detection rate was 85.29%.

The calculated results demonstrated that nodes in the hippocampus dominated either in the pre-ictal or in the early-ictal period and nodes within the HH showed prominent results in the pre-ictal at 5 min (Figures 3C,D). We interpreted these results as an indication of hippocampal involvement and as a functional state of the remaining HH in the seizure network. The calculated out-degrees indicated the influenced range of a single neural population. This included populations within the remaining HH that would process less out-degrees for the outputs of the HH and were fewer than those of the hippocampus. Whether this method overcame the relatively low potential induced by the exclusion of the majority of the lesion needs to be further studied. One future problem to solve is how to reduce the interference from the signal *per se*. In addition, the pre-ictal appearance of the prominent HH out-degree and dominant hippocampus out-degree could be interpreted as the hippocampus having become the main functional unit, and the HH might work as a pacemaker. A recent *in vivo* study showed that the loss of neuronal network resilience in the inter- and pre-ictal period might precede seizures (28), which indicated the importance of inter- and pre-ictal periods. Furthermore, the nodes within the white matter were not excluded. Theoretically, electrical activities of white matter are secondary to those of the neural populations, and the involvement of the white matter does not change the results generated by the whole network.

There were some limitations to the study, which could not be resolved by the present techniques. Besides the previously mentioned signal bias, the low coverage rate of SEEGs might omit important neural populations only by non-detection, and the method still has to be explored in larger clinical studies.

In conclusion, the importance of this case was that not only the existence of the dependent stage of secondary epileptogenesis was verified, but also the ability of computational methods to reveal information that could not be manually interpreted was demonstrated. In clinical processes, secondary epileptogenesis needs to be considered, and in the future, computational methods might suggest novel diagnoses and treatment of epilepsy.

## Data Availability Statement

All datasets generated for this study are included in the article/supplementary material.

## Ethics Statement

The studies involving human participants were reviewed and approved by The Ethics Committee of Sanbo Brain Hospital, Capital Medical University. Written informed consent was obtained from the minor(s)' legal guardian/next of kin for the publication of any potentially identifiable images or data included in this article.

## Author Contributions

ZL collected the patient information, conceptualized and designed the study, performed the SEEG-guided thermocoagulation, and drafted the manuscript. CY and QW conducted the computational calculations. MW and JW performed the clinical data analyses. GL, YG, and CL conducted the electrode implantations.

## Conflict of Interest

The authors declare that the research was conducted in the absence of any commercial or financial relationships that could be construed as a potential conflict of interest.
